# Oxidized Monolayers of Epitaxial Silicene on Ag(111)

**DOI:** 10.1038/srep22510

**Published:** 2016-03-03

**Authors:** Neil W. Johnson, David I. Muir, Alexander Moewes

**Affiliations:** 1 University of Saskatchewan, Department of Physics and Engineering Physics, Saskatoon, S7N 5E2, Canada; 2 Canadian Light Source, Saskatoon, S7N 2V3, Canada

## Abstract

The properties of epitaxial silicene monolayers on Ag(111) at various levels of oxidation are determined through complementary density functional theory calculations and soft X-ray spectroscopy experiments. Our calculations indicate that moderate levels of oxidation do not cause a significant bandgap opening in the epitaxial silicene monolayer, suggesting that oxygen functionalization is not a viable mechanism for bandgap tuning while the silicene monolayer remains on its metallic substrate. In addition, moderate oxidation is calculated to strongly distort the hexagonal Si lattice, causing it to cluster in regions of highest oxygen adatom concentration but retain its 2D sheet structure. However, our experiments reveal that beam-induced oxidation is consistent with the formation of islands of bulk-like SiO_2_. Complete exposure of the monolayer to ambient conditions results in a fully oxidized sample that closely resembles bulk SiO_2_, of which a significant portion is completely detached from the substrate.

Freestanding silicene monolayers (FSMLs) are expected to exhibit many of the same electronic characteristics of their carbon-based counterpart, graphene^1^,^2^,^3^. This includes hosting charge-carrying quasiparticles that behave as massless Dirac fermions, with the Fermi velocity in FSMLs calculated to be approximately half that of graphene^4^. The buckled structure of FSMLs combined with the large spin-orbit coupling in Si may lead to properties that currently cannot be achieved by single-layer graphene, such as bandgap^5^,^6^ and spin-polarization^6^ control through the application of a gating voltage. These characteristics have led to considerable interest in silicene-based electronic and spintronic devices. However, truly freestanding silicene is yet to be observed. The propensity for Si to assume the *sp*^3^ hybridization scheme prevents it from forming a stable graphite-like layered structure, so silicene cannot be derived directly by exfoliation and must instead be constructed in a bottom-up manner via deposition on a supporting substrate. These epitaxial silicene monolayers (ESMLs) were initially observed on the Ag(111) face^7^, and have since been reported on Ir(111)^8^ and ZrB_2_(0001)^9^. Further, it has been predicted that stable ESMLs could exist on SiC(0001)^10^, h-BN^10^,^11^ and Pb(111)^12^. In the case of ESMLs on Ag(111), the most thoroughly studied of the ESML systems, an unexpectedly strong interaction with the underlying substrate has been shown to induce semi-metallic character in the silicene^13^,^14^,^15^,^16^. In the absence of a substrate, these ESMLs are expected to once again become semiconducting^16^,^17^, but the Ag(111) crystals that play host to the silicene are often too large and expensive to make substrate etching a technically feasible or economical option.

Recently, the first functioning transistor based on an ESML was reported^18^. An ultra-thin Ag(111) film deposited on a mica substrate acted as a growth template for the silicene. The ESML was then capped with Al_2_O_3_ and transferred to a silicon wafer along with the Ag(111), which had been mechanically separated from the mica. Due to the thinness of the Ag(111) film, etching it away was a viable method for exposing the bare ESML. It exhibited a brief period of transistor behaviour before becoming completely oxidized and losing functionality. The creation of such a device marks a significant step forward in silicene-based technology, but its short lifetime (on the order of a few minutes) in ambient conditions indicates that understanding and preventing the oxidation of ESMLs will be of great importance to further advances in this field. Controlled oxidation has been suggested as a possible manner in which a the bandgap of FSMLs could be modulated^19^, but it is currently not clear how this affects the properties of ESMLs^20^,^21^.

In this study, we consider the effect of oxidation on the prototypical 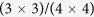 ESML on Ag(111). Using all-electron, full-potential density functional theory (DFT) refinements of the atomic structure, we show that these ESMLs are capable of dissociating O_2_ molecules and that the O atoms prefer Si–Si bridging sites. Our calculations predict that the hexagonal structure of the ESML becomes highly distorted in the process of oxidation, but also indicate that the sheet will maintain a significant interaction with the underlying substrate and retain its semi-metallic nature. Through soft X-ray emission and absorption spectroscopy (XES and XAS, respectively) at the Si L_2,3_ edge, we show that controlled, beam-facilitated oxidation is achievable. However, this does not result in a homogeneously oxidized ESML like that predicted by DFT, instead resembling islands of bulk-like SiO_2_ within an otherwise unaffected ESML.

## Results

### DFT Structure Optimization and Electronic Structure Calculation

**Figure 1:**
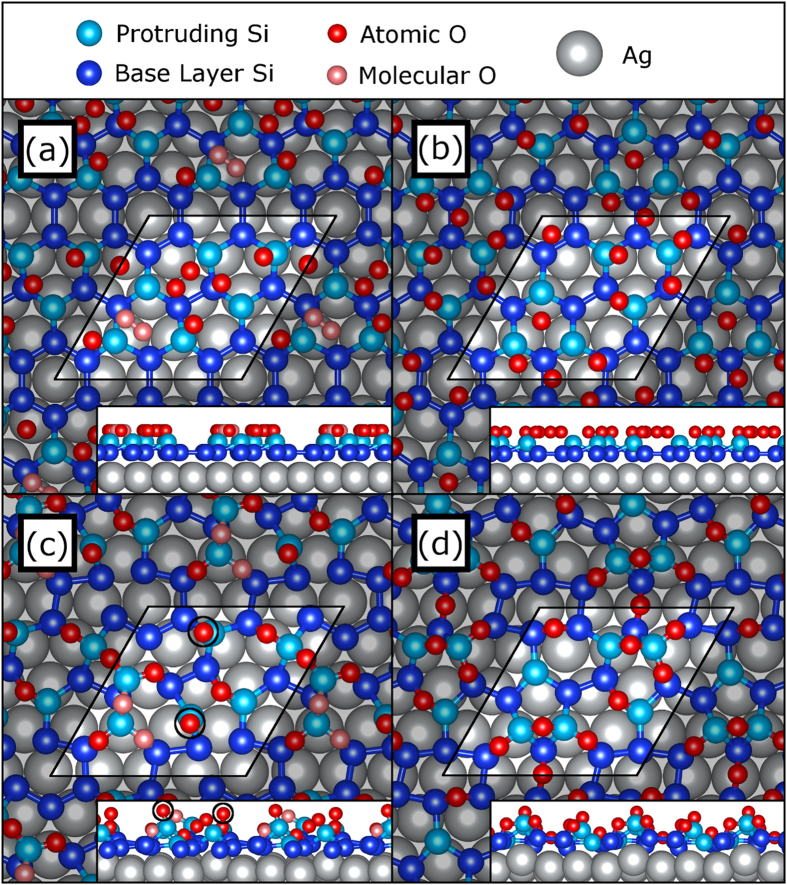
(**a**,**b**) Initial configurations of the oxidized 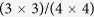 silicene/Ag(111) system prior to structural relaxation, with (**a**) a mix of O atoms shown in dark red and an O_2_ molecule shown in light red, and (**b**) 9 O atoms. Initially protruding Si sites are shown in light blue and initially base-layer sites are shown in dark blue, while the Ag(111) substrate is depicted as silver spheres. The highlighted window shows the unit cell of the calculation, which contains a 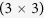 silicene cell commensurate with a 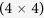 Ag(111) template. (**c**,**d**) Final configurations of (**a**,**b**), respectively. The dissociation of the O_2_ molecule is visible in (**c**), despite the calculation not reaching force convergence due to the atoms circled in black. In (**d**), the entire structure has reached force convergence. Visualization provided by the VESTA software package^30^.

Specific details regarding the DFT calculation parameters are mentioned in the Methods section below. Previously^16^, we noted a strong similarity between the electronic structures of the prototypical 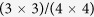 silicene/Ag(111) configuration and the 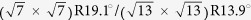 silicene/Ag(111) configurations that are usually observed simultaneously in ESML samples. For this reason, we only consider the 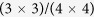 superstructure in this oxidation study, since the results can likely be generalized to the other stable ESML structures. This structure is composed of 18 Si atoms per 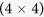 Ag(111) supercell, with 12 of the Si sites located on the base layer and 6 Si sites protruding from it, as shown in Fig. 1(a,b). Each of the three structures considered in this study are initialized with nine O sites randomly scattered in a plane 1.5 Å above the topmost sheet of Si atoms, representing a coverage of 0.5 monolayers. Where the randomly generated O coordinates overlap, the first set of overlapping O atoms are separated to the O_2_ bond distance (1.21 Å) and subsequent overlaps are resolved by selecting new random coordinates for one of the atoms. Two of the three structures generated in this manner contain one O_2_ molecule, while one contains only atomic O sites. The initial configurations of an O_2_ molecule-containing structure and the purely atomic O structure are shown in Fig. 1(a,b), respectively.

Through force relaxation, the O_2_ molecule-containing ESML eventually evolves to the structure shown in Fig. 1(c). It is not found to become fully relaxed after a few hundred iterations, as two of the O atoms (highlighted with black circles in Fig. 1(c)) still experience forces larger than the convergence criterion. These atoms appear to have found a metastable force minimum directly above a protruding Si site, which they oscillate in as the surrounding lattice continues to readjust. We do not expect such O configurations to be physical as these calculated structural relaxations neglect the effects of thermal energy, which would likely allow the O atoms greater mobility over the Si lattice. However, as the molecular O_2_ (light red in Fig. 1(a,c)) is observed to fully dissociate and settle into the silicene lattice, we can conclude that ESMLs are capable of dissociating atmospheric O_2_, even at zero temperature. This has previously been shown for FSMLs^22^, but to our knowledge this is the first theoretical observation of O_2_ dissociation on ESMLs in the literature. Going forward, we will not consider the electronic structure of this particular oxidized ESML as it is not fully relaxed.

The ESML initiated with a plane of atomic O is found to completely relax below the convergence criterion, resulting in the structure shown in Fig. 1(d). Each of the O atoms penetrates the silicene lattice and forms a bridge between adjacent Si atoms, in a Si–O–Si bond somewhat like those found in silica. However, the Si–O bond lengths (ranging from 1.65 Å to 1.74 Å) are slightly longer and the Si–O–Si bond angles (ranging from 97.0° to 122.5°) are much sharper than the distribution of values typically found in silica^23^. Si atoms are observed to bond with anywhere from zero to three O atoms, forming sites that will henceforth be referred to as unoxidized, single-bridge, double-bridge and triple-bridge sites. Unoxidized and single-bridge Si atoms typically lie closer to the Ag substrate than the double-bridge and triple-bridge sites.

We find that the presence of 9 O atoms per unit cell strongly distorts the hexagonal silicene lattice, with Si–Si bond lengths ranging from 2.30 Å to 2.98 Å compared to the bond lengths of 2.28 Å to 2.31 Å in unoxidized 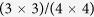 ESMLs. The distortion manifests as a bunching of the Si atoms in the regions of highest O atom concentration and a corresponding stretching of the lattice in regions with fewer O atoms. In our simulations, oxidation does not promote any of the initially base layer silicene atoms to protruding silicene atoms, but some of the initially protruding atoms are pulled down to the base layer through local stretching. Further, the overall thickness of the ESML (calculated as the vertical distance between the lowest and highest Si atoms) increases from 0.75 Å for the unoxidized ESML to 2.20 Å for the oxidized ESML, in which the triple-bridge site has the highest vertical position.

A previous study performed by Du *et al.*^20^ examined epitaxial silicene through DFT and scanning tunnelling microscopy (STM) in the weakly oxidized regime, at O concentrations of one or fewer atoms per unit cell. The authors found through DFT calculations that single O atoms tend to bridge protruding and base layer Si sites, pulling the protruding atom down to the base layer. In contrast, we find that oxidation of the protruding sites tends to increase their height above the Ag substrate, while the unoxidized protruding sites migrate into the base layer. We see instances of O atoms residing in protruding–protruding, protruding–base and base–base bridge sites at this level of oxidation.

Our calculations closely resemble the pseudopotential calculations of Xu *et al.*^21^, who performed a similar structural optimization except that their initial O configuration appears not to have been randomized, instead being deliberately initialized either directly over Si sites or in Si-Si bridging positions. The authors suggest that the distortion observed in the ESML lattice is evidence for decoupling from the underlying Ag(111) substrate. However, their calculations as well as ours show that the structure of the underlying Ag(111) substrate is strongly perturbed by the reorganization of the ESML resulting from oxidation, suggesting that ESML/substrate interactions are still significant even in the presence of 9 O atoms per unit cell.

**Figure 2:**
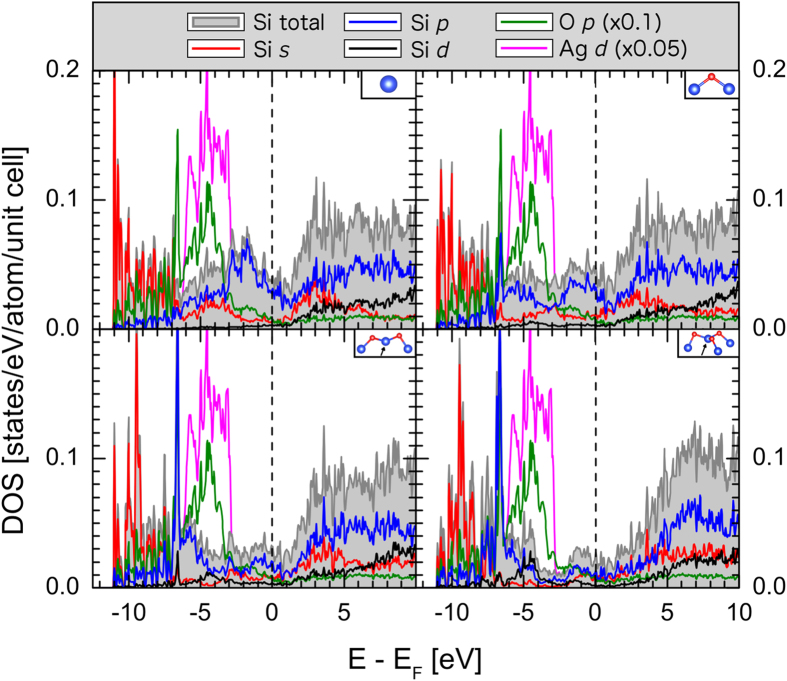
Partial and total Si DOS of the fully relaxed ESML initialized with atomic O are shown in solid lines, separated by the number of O bridge sites attached to the Si atom (see inset model). The *p* character of the O atoms in this model is shown in dotted lines in order to illustrate the Si *p*–O *p* hybridization peak that occurs at −6.7 eV.

The partial Si densities of states (pDOS) for the converged calculation are shown in Fig. 2(a–d), separated by the number of O atoms bonded to each Si site. Unoxidized Si atoms bear a strong electronic similarity to the net pDOS of unoxidized ESMLs that we presented in a previous publication^16^, especially in terms of the Si *p* states. For example, there is a strong presence of Si *s* and *p* states spanning the Fermi level, indicative of hybridization with the Ag *sp* band in that region. In addition, there appears to be significant hybridization between the unoxidized Si *s* and *p* valence states and the Ag *d* states in the energy range of −7 eV to −3 eV. A number of trends emerge with the addition of more O bridging bonds. Among them, a noticeable weakening of the number of Si *p* states at the Fermi level, while the contribution from Si *s* and *d* states stay approximately constant. The majority of these *p* electrons move to the range of −8 eV to −5 eV relative to the Fermi level, which corresponds to Si *p*–O *p* hybridization, evident from the strong O *p* peak also located at that energy. Oxidation compresses most of the Si *s* states into a sharp feature ranging from the bottom of the valence band (VB) to −8 eV while the contributions elsewhere in the VB are weakened and moved toward the VB maximum. Notably, oxidation also is observed to push states away from the Ag *d*-dominated region, implying a significant weakening of direct hybridization with the substrate. This is to be expected, as oxidation introduces a large spatial separation between the Si atoms from the Ag substrate. In the case of double- and triple-bridged Si sites, there is a significant density of Si *d* states introduced into the valence band, mostly between −8 eV and −3.5 eV relative to the Fermi energy. These states also show signs of O *p* hybridization, much like the Si *d*–O *p* hybridization observed in alpha quartz^24^. In the conduction band (CB), the primary effect of oxidation seems to be an energetic shift upwards of features in the *s*, *p* and *d* pDOS, as well as an overall softening of their onset. In general, changes to the VB are much more pronounced than those of the CB under the addition of oxygen.

Finally, the calculations indicate that even the triple-bridge Si sites do not possess a bandgap, though the DOS in the vicinity of the Fermi level is significantly reduced. This finding contradicts the scanning tunnelling spectroscopy (STS) observation of a local bandgap of 0.1 eV to 0.3 eV with an oxygen exposure of 20 L (approximately 3 nm between adjacent O adatoms)^20^, and indicates that the oxidized ESML remains semi-metallic even at this heavier level of oxidation. This prediction verifies the DFT calculations performed by Xu *et al.*^21^, in which the lack of a bandgap was also observed in the DOS.

Soft X-ray spectroscopy at the Si L_2,3_ edge is sensitive to the Si *s* and *d* pDOS in the VB and CB, as it relies on monitoring radiative 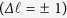 transitions to and from the Si 2*p* core level. While it might be preferable to probe the Si *p* states directly by conducting Si K-edge soft X-ray spectroscopy, as the *p* states are responsible for the unique electronic characteristics of silicene, the penetration depth of the X-rays required to create a Si 1*s* core hole is too large to achieve a useable level of signal from a monolayer Si sample. In the following sections, we discuss the growth and oxidation of an ESML sample on Ag(111) and characterize the electronic changes induced by oxidation using soft X-ray emission and absorption spectroscopy (XES and XAS, respectively) at the Si L_2,3_ edge.

### Soft X-ray Spectroscopy

**Figure 3:**
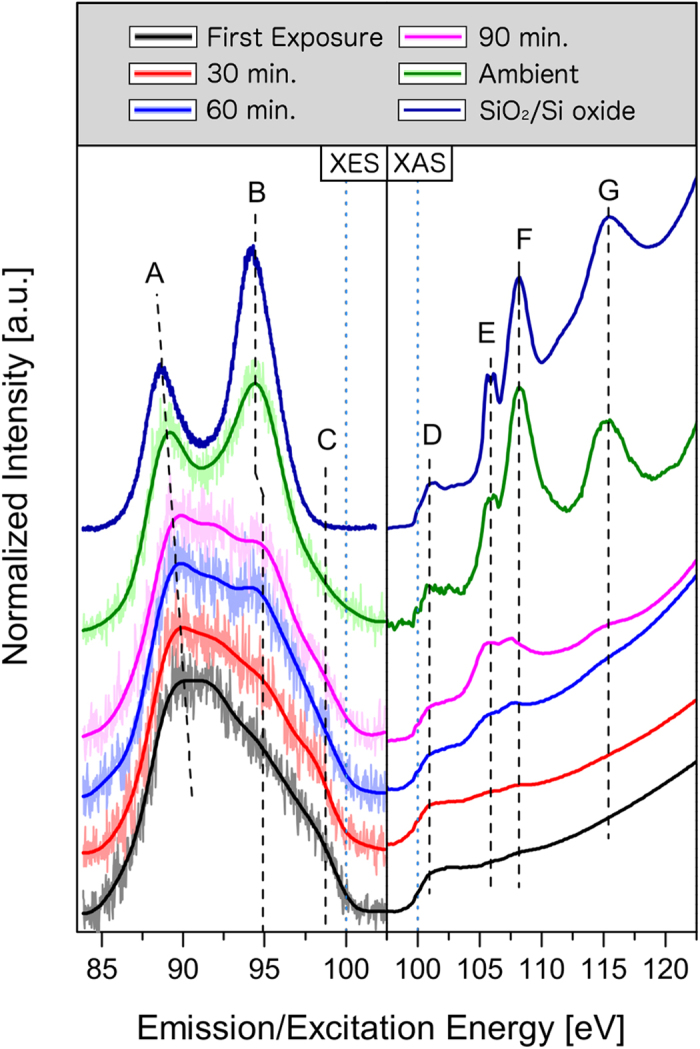
Si L_2,3_ XES (left pane) and TEY-mode Si 2*p* XAS (right pane) measurements of ESMLs at various levels of oxidation, with a 15-point FFT smoothing overlaid on the monolayer XES data for clarity. Spectra obtained after the *in vacuo* transfer are labelled by the total amount of XES beam exposure prior to the measurement, while the fully oxidized ESML is labelled “Ambient”. Also included is the emission spectrum of bulk SiO_2_ and the absorption spectrum of a native surface oxide on a Si wafer for reference. Vertical blue dotted lines indicate 100 eV on both panes to show approximately where the XES/XAS overlap occurs.

Details regarding sample synthesis and obtaining soft X-ray spectra are provided in the Methods section. We begin by examining the XES spectra of the ESML, presented in the left panel of Fig. 3 along with their smoothed profiles for clarity. The first emission spectrum, taken directly after transfer and the first XAS scan, has the somewhat featureless, wedge-shaped profile associated with ESMLs^16^. It consists of a broad emission peak around 90 eV, a very weak shoulder around 94.9 eV and the high-energy shoulder at around 98.5 eV that represents the Si *s* and *d* states near the VB maximum. Subsequent emission measurements show a separation of the main emission feature into two components – one fixed at around 91.7 eV and one that migrates downward with beam exposure (“A”). The very weak feature at 94.9 eV (“B”) grows in strength with each consecutive scan, while the VB maximum (“C”) decreases in spectral weight but does not appear to shift appreciably in energy. In a previous study of ESMLs, we hypothesized that this was a result of beam-induced oxidation as the peak at 94.9 eV agrees well with the main emission feature of SiO_2_, also shown in the left panel of Fig. 3. Indeed, when the ESML has been fully exposed to ambient conditions, the resulting XES spectrum strongly resembles that of SiO_2_, aside from a slight energetic shift of the lower peak, a difference in A:B peak ratios and a higher VB maximum with a broader tail.

The XAS is initially fairly featureless, only possessing a strong edge jump (“D”) similar to that of the absorption spectrum of bulk Si, attributed to the CB onset^16^. Some very weak peaks are apparent at 105.8 eV and 108.1 eV (“E” and “F”), which are found to grow in intensity with continued beam exposure. With 1 hr of accumulated XES exposure, another feature is observed to emerge around 115.3 eV (“G”). Again, each of these features correspond well to those observed in the native surface oxide on a Si wafer, also shown in the right panel of Fig. 3. In fact, when the sample is fully exposed to air, the resulting XAS spectrum is virtually identical to that of the native surface oxide on a Si wafer in terms of peak energies and ratios.

**Figure 4:**
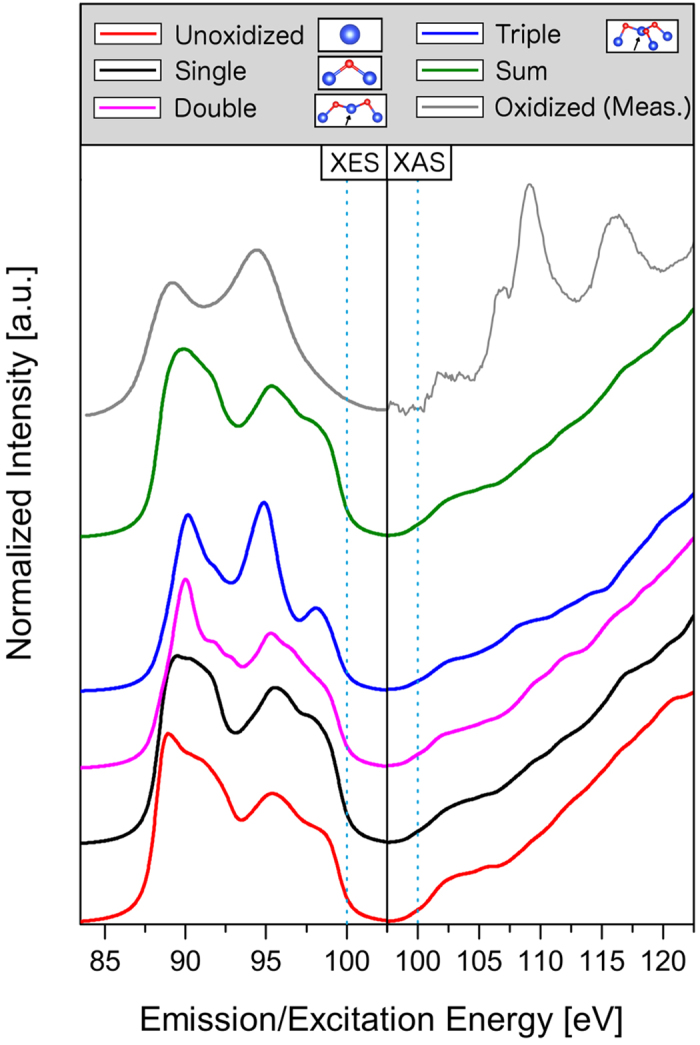
Calculated Si L_2,3_ XES and Si 2*p* XAS spectra for the oxidized ESML model, separated by number of O bridging sites. Vertical blue dotted lines again indicate 100 eV to facilitate comparison with the measured spectra, of which the fully oxidized spectra are also shown here (grey, XES smoothed).

The WIEN2k utility XSPEC, which is based on the formalism described in ref. 25, is used to generate calculated Si L_2,3_ XES and Si 2*p* XAS spectra from the Si pDOS, which are shown in the left and right panels of Fig. 4 respectively. Spectra corresponding to unoxidized, single-bridge, double-bridge and triple-bridge sites are displayed individually, along with the weighted sum of these spectra representing the total XES and XAS spectra resulting from the whole sample and the fully oxidized measurements for comparison. Generally, the XES spectra show only minor changes from zero to two bonded O atoms, including a slight shift of the lowest energy feature from 88.7 eV to 90.0 eV as well as a narrowing of that same feature. However, with the third O bridge in place, the spectrum changes considerably, having three distinct peaks at 90.0 eV, 94.7 eV and 98.2 eV. The second peak corresponds closely to the feature in the XES spectra of the oxidized ESML that increases with beam exposure. However, were this the dominant mechanism for beam-induced oxidation in the ESML, the observed downward tracking of feature A would not be expected to occur and the weakening of the VB maximum region should not be as evident. The XES data are better explained as the direct sum of unoxidized silicene and bulk-like SiO_2_ spectra than as the oxidized ESML derived through DFT structural optimization.

The calculated XAS spectra differ significantly from the measured spectra in that the absorption onset is much smoother in the calculations. This implies that the concentration of Si *s* and *d* states in the lower CB is larger in the experimental samples than the model predicts. Weak features are found at 105.6 eV in the single-bridged Si site, 109.5 eV and 112 eV in the double-bridged site, and 108.2 eV in the triple-bridged site. Only the single-bridged site feature corresponds well to a peak in the experimental oxidized ESML spectrum, but fails to adequately describe the entire absorption profile. Again, we draw the conclusion that the XAS indicates regions of bulk-like SiO_2_ formation instead of an oxidized sheet of silicene.

Together, these spectral changes with beam-induced oxidation followed by complete ambient exposure indicate that the dominant mechanism by which ESMLs oxidize is the growth of bulk-like SiO_2_ islands in an otherwise unoxidized monolayer. This mechanism explains the observation that the XES spectra appear to be the direct summation of an ESML spectrum and the SiO_2_ spectrum, weighted by beam and oxygen exposure. It also explains the evolution of the XAS spectra with increasing oxidation. One thing that becomes apparent under this interpretation is that, even after 10 minutes of ambient exposure, there is still a detectable trace of ESML-like features in both the XES and XAS spectra. This includes the slightly higher energy of feature A, the A:B peak ratio that does not agree with bulk SiO_2_, the presence of states around the ESML VB maximum at C, and the bulk Si-like absorption onset D, which is absent in the XAS spectrum of pure SiO_2_^26^ but present in that of the native oxide on a Si wafer.

Another observation worth noting is the decreased signal strength observed in both the XES and XAS measurements after complete ambient exposure of the ESML. The count rate for the XES was approximately halved, and the noise level in the XAS spectrum increased appreciably. This occurred uniformly across the sample surface. That is, there were no spatial regions of increased count rate to balance the loss of counts elsewhere. A likely interpretation of this observation is that the complete oxidation of the ESML causes regions of the sample to flake off of the substrate, resulting in less overall Si on the surface.

Were oxidation to open a bandgap in the experimental samples, it would manifest as a reduction in overlap of the XES and XAS, or even a visible break between the two spectra if the gap exceeds the broadening introduced by instrumentation, core hole lifetime and Si 2*p*_1/2_–2*p*_3/2_ core-level splitting. While it is difficult to pinpoint the exact energy of the VB maximum owing to the weakness of Si *s* and *d* states in the upper VB, we see no convincing evidence of a bandgap opening in our measured spectra, indicating the continued existence of a semi-metallic or metallic Si species on the surface even after complete oxidation.

It could be argued that beam-induced oxidation is not a good analogue for controlled oxidation through low-level O_2_ gas exposure, as soft X-ray beams can deposit a significant amount of energy into a material, breaking bonds in the sample and increasing its temperature. However, in the multiple oxidation experiments that we carried out (see Supplemental Information), samples that were transferred under higher pressure conditions had initial XES and XAS spectra that looked virtually identical to the reported sample’s spectra after one or two cycles of XES and XAS measurements. That is, the spectra looked the same regardless of whether the sample had been oxidized by beam exposure or by exposure to higher pressures (and therefore more O_2_ content) during the transfer process. This allows us to conclude that the soft X-ray beam simply accelerates the oxidation process and does not represent an entirely different oxidation mechanism from atmospheric exposure to O_2_.

## Discussion

DFT calculations indicate that ESMLs are capable of dissociating atmospheric O_2_, with O atoms tending toward Si–O–Si bridge sites during oxidation. At a concentration of 9 O atoms per unit cell, the oxidized ESMLs are predicted to remain locally semi-metallic even at Si sites that possess three Si–O–Si bridge bonds. Oxidation causes the degradation of the hexagonal Si lattice, with Si sites clustering to the regions of highest O concentration and retracting elsewhere, and bonding with O atoms is found to draw Si sites upward out of the initial monolayer. This prediction is in contrast to the STS data that suggest a bandgap opening of 0.1 eV globally and 0.3 eV locally in weakly oxidized silicene sheets^20^. Structural relaxations also indicate that the substrate is significantly distorted by the oxidation of the ESML, suggesting that the substrate/ESML interaction is still significant even at a 2:1 Si:O ratio.

Experimentally, moderately oxidized ESMLs on Ag(111) are confirmed as retaining the semi-metallic nature of pristine ESMLs, suggesting that oxidation is not a suitable technique for bandgap modulation while the ESMLs remain on their substrate. Further, soft X-ray beam-induced oxidation and exposure to ambient conditions are both found to generate regions of bulk-like SiO_2_ rather than evenly coating the ESML with adsorbed O. Ambient exposure is also found to cause some of the ESML to slough off of the substrate, likely in the form of very small, condensed SiO_2_ crystals.

The disagreement between the theoretical and experimental oxidized structures is a result of the inability of DFT to properly model the dewetting of the Si sheet into SiO_2_-like bulk crystals. DFT is constrained to produce a relaxed structure that conforms to the periodicity requirements of the unit cell, which prevents it from predicting the formation of bulk clusters that are much larger than a single cell. Further, DFT calculations occur at zero temperature and are therefore prone to converge at local energy minima and not the global energy minimum. It is therefore a distinct possibility that the DFT-predicted structure could be stable at low temperatures, but not at room temperature. However, our theoretical results are still valuable in that they demonstrate that a 2D oxidized structure cannot reproduce our XES and XAS spectra to the same degree as a model consisting of bulk-like SiO_2_ and unoxidized silicene, and they allow us to characterize a 2D oxidized structure that may yet be produced, even if it is only stable at low temperatures.

While oxidation of FSMLs and ESMLs that have been etched from their Ag(111) substrates may result in silicene with tuneable electronic characteristics, complementary DFT calculations and soft X-ray spectroscopy measurements indicate that moderately oxidized ESMLs on Ag(111) retain the semi-metallic nature of their unoxidized counterparts, and fully oxidized ESMLs on the same substrate tend to crystallize into regions of bulk-like SiO_2_. This result has significant implications toward the production of silicene-based electronics, which in the absence of a passivating substance are likely to experience atmospheric O_2_.

## Methods

### DFT Calculations

As in previous studies^16^,^27^, we use the “slab method” to approximate the two-dimensional ESMLs in a way that satisfies DFT’s requirement for a periodic three-dimensional structural input. The ESMLs are modelled as Si sheets on both faces of a five unit cell thick slab of Ag(111) with 15 Å of vacuum inserted between adjacent unit cells in the direction normal to the ESMLs. This results in a layered structure consisting of slabs of Si-coated Ag extending infinitely in the a and b crystallographic directions separated from neighbouring slabs by a large vacuum gap. Along with the choice of a single k-point in the c direction, this gap prevents a spurious electronic interaction between adjacent silicene sheets.

All DFT calculations are performed using the *ab initio* all-electron LAPW + lo WIEN2k software suite^28^, using the generalized gradient approximation of Perdew, Burke and Ernzerhof (PBE-GGA)^29^. The plane-wave cutoff energy is −6 Ry, and the k-point mesh and RK_*max*_ are set to 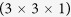 and 5 respectively for the structural relaxation and 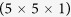 and 6 respectively for the electronic structure calculations. Structural relaxations are considered converged when the net force calculated for each mobile atom (the middle plane of Ag atoms are fixed at their bulk Ag positions) falls below 1 mRy/au, and all calculations are considered converged when charge and energy steps fall below 10^−3 ^e and 10^−4 ^Ry, respectively.

### Sample Preparation and Soft X-ray Spectrsocopy

ESMLs were synthesized through the physical vapour deposition of a Si wafer onto a 250 °C single-crystal Ag(111) disk in an ultra-high vacuum (UHV) preparation chamber (base pressure 10^−9 ^Torr). Prior to deposition, the disk surface was prepared with two cycles of annealing at 500 °C and sputtering for 1 hr by 1 keV Ar^+^ ions at an Ar pressure of 10^−6 ^Torr. Deposition of a monolayer lasted 35 minutes with a Si current of 12 A and a source-to-substrate distance of about 15 cm. The deposition was performed at the Resonant Elastic and Inelastic X-ray Scattering (REIXS) beamline of the Canadian Light Source (CLS).

The monolayer nature of the sample was confirmed with low-energy electron diffraction (LEED), as in our previous studies^16^,^27^. As expected under these growth conditions, the sample was observed to contain a mixture of 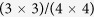 and 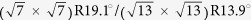 character. The ESML was then transferred to the soft X-ray spectroscopy measurement chamber via a high-vacuum (HV) transfer chamber with a base pressure of 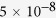 Torr. Prior to measurement the total exposure of the sample was approximately 90 L. After all measurements on the fresh ESML were performed, the sample was then removed from the measurement chamber and exposed to ambient conditions for 10 minutes in order to create a fully oxidized ESML, which was then reinserted into the UHV chamber for additional soft X-ray spectroscopy measurements.

All XAS spectra are calibrated such that the main absorption feature of the native oxide on a Si wafer (labelled “F” in 3) occurs at 108.1 eV, while the emission spectra were calibrated using elastic scattering peaks so that they are consistent in scale with the monochromator and therefore the XAS measurements. The monochromator resolving power was  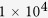 at the Si 2*p* absorption edge, while the emission spectrometer, which uses diffraction gratings in a Rowland circle geometry as dispersive elements and is fitted with a microchannel plate detector, had a resolving power of  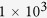 at the Si L_2,3_-edge. For each spot on the sample surface, a 20 minute XAS scan was performed in surface-sensitive total electron yield (TEY) mode with the exit slits at 25 μm, followed by a 30 minute XES exposure with a 200 μm exit slit. This XAS/XES cycle was then repeated three times per sample spot, in order to capture any changes to the electronic structure that may arise as a result of beam exposure. As the slit size and subsequent dose is much larger for XES measurements than XAS, any beam-induced changes to the sample should happen primarily during the XES measurements. Measurements are therefore grouped by the cumulative XES exposure prior to the spectrum being obtained.

## Additional Information

**How to cite this article**: Johnson, N. W. *et al.* Oxidized Monolayers of Epitaxial Silicene on Ag(111). *Sci. Rep.* **6**, 22510; doi: 10.1038/srep22510 (2016).

## Supplementary Material

Supplementary Information
