# Supplementary material for: Oxidized Monolayers of Epitaxial Silicene on Ag(111)
Source: Sci Rep. 2016 Mar 3;6:22510. doi: 10.1038/srep22510 (PMC4776149; doi:10.1038/srep22510)
Supplement: Supplementary Information [file srep22510-s1.pdf]

<u>Supplementary Information</u>

"Oxidized Monolayers of Epitaxial Silicene on Ag(111)"

Neil W. Johnson[1], David I. Muir[2] and Alexander Moewes[1]
[1] University of Saskatchewan, Department of Physics and Engineering Physics, Saskatoon, S7N 5E2, Canada
[2] Canadian Light Source, Saskatoon, S7N 2V3, Canada

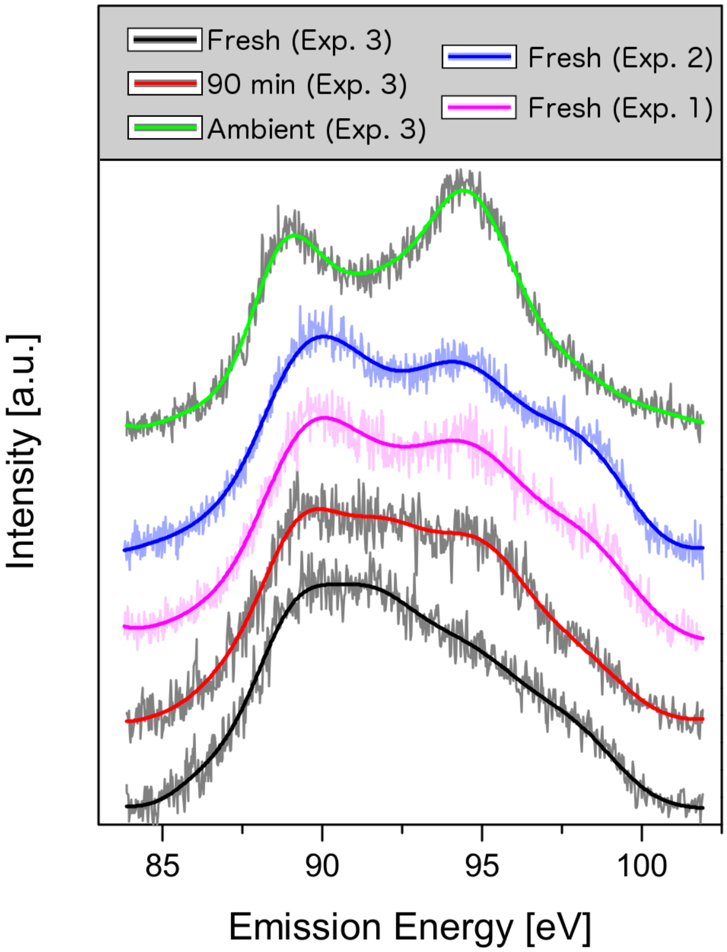

Figure S1: The Si $L_{2,3}$ XES spectra (and smoothed curves) for three separate monolayer silicene samples. Experiment 3 (gray unsmoothed spectra) had the lowest exposure to oxygen prior to measurement, and its spectra are shown for initial exposure to the synchrotron beam, 90 minutes of exposure to the beam and after being exposed to ambient conditions. Experiments 2 and 3 were synthesized, transferred and measured at higher pressures, resulting in pre-measurement oxidation and its characteristic XES feature around 95 eV.
